# Stress-Related Immune Response and Selenium Status in Autoimmune Thyroid Disease Patients

**DOI:** 10.3390/ijms24032440

**Published:** 2023-01-26

**Authors:** Ieva Vaivode, Tatjana Zake, Ieva Strele, Sabine Upmale-Engela, Deniss Gogins, Gita Gersone, Andrejs Skesters, Maija Dambrova, Ilze Konrade

**Affiliations:** 1 Department of Internal Diseases, Riga Stradins University, LV-1007 Riga, Latvia; 2 Institute of Occupational Safety and Environmental Health, Riga Stradins University, LV-1007 Riga, Latvia; 3 Department of Human Physiology and Biochemistry, Riga Stradins University, LV-1007 Riga, Latvia; 4 Latvian Institute of Organic Synthesis, LV-1006 Riga, Latvia

**Keywords:** human health, autoimmune thyroid disease, selenoproteins, glutathione peroxidase, stress, cytokines

## Abstract

Autoimmune thyroid disease (AITD), including Graves’ disease (GD) or Hashimoto’s thyroiditis (HT), occurs due to genetic susceptibility and environmental factors, among which the role of stressful events remains controversial. This study investigated the relationship between the number and impact of stressful life events in AITD patients with selenium status, and the Th1/Th2/Th17 immune response. The study population included three groups: HT (*n* = 47), GD (*n* = 13), and a control group (*n* = 49). Thyroid function parameters, autoantibody levels, and the plasma levels of cytokines, selenium, selenoprotein P (SeP), and glutathione peroxidase 3 (GPx) activity were measured. Participants filled out the Life Experiences Survey. No significant differences in the number of stressful life events were found among the patients with HT, GD, and the controls. A higher (median (interquartile range)) negative stress level (8 (4–12)) than a positive stress level (3 (1–9)) was found in the HT group. The HT group showed a correlation between SeP and the positive stress level: r_s_ = −0.296, *p* = 0.048, and the GD group between GPx and the negative stress level (r_s_ = −0.702, *p* = 0.011). Significant positive correlations between thyroid peroxidase antibody level and the total number of major life events (*p* = 0.023), the number of major life events in the last 7–12 months, and the number of major life events with no impact and a negative stress level were found. We suggest that the measurements of Th2-related cytokines and selenoproteins could be used as biomarkers for the development of AITD in cases where stress is considered a component cause of the pathogenic mechanism of the disease.

## 1. Introduction

Autoimmune thyroid disease (AITD), including Graves’ disease (GD) or Hashimoto’s thyroiditis (HT), occurs due to an interaction between genetic susceptibility, which contributes approximately 70–80% in the development of thyroid autoimmunity, and various environmental factors, such as increased iodine intake, selenium deficiency, smoking status, alcohol consumption, infections, drug side effects, microbiota, and stressful life events [1,2,3]. Numerous studies on psychological stress have been inconclusive [4,5,6,7,8], but several excellent mechanism-based studies have examined the pathways the immune deviation [9,10,11] and have named stress as an exacerbating factor for autoimmune disease activity. Stress plays an important role in the response to therapy [12,13,14], and perhaps, in the aetiology as well [15,16].

HT and GD have distinct and predominant T helper (Th) 1- and Th2-mediated immune responses, respectively [17,18]. Thus, GD is characterised by increased levels of Th2-released cytokines, such as interleukin (IL)-4, IL-5, IL-6, and IL-13, which mainly mediate the humoral response-induced production of autoantibodies to the thyroid-stimulating hormone (TSH) receptor. Whereas in HT, the upregulation of Th1 cytokines, such as IL-2, IL-1β, interferon (IFN)-γ, and tumour necrosis factor (TNF)-α, leads to cell-mediated immunity and causes thyroid cell apoptosis [19]. Recently, it was discovered that Th17 cells also play a role in the pathogenesis of thyroid autoimmunity [20,21,22].

Regardless of the early paradigm that considered the stress-related effects as primarily immunosuppressive, more recent findings provide evidence that both acute and chronic stress might affect the homeostasis of the human immune system and induce the development of an autoimmune disease. During stress, activation of the hypothalamic-pituitary-adrenal axis and the sympathoadrenal system causes an oversecretion of glucocorticoids and catecholamines, which both have a similar immunoregulatory effect and induce the Th1/Th2 imbalance characteristic of GD. Stress hormones suppress IL-2 production by antigen-presenting cells and enhance the secretion of IL-4 and IL-10 by Th2 cells, thereby directing immunity towards Th2 responses [9,10,23]. In addition, glucocorticoids inhibit the synthesis of IL-1, IL-2, TNFα, and IFNγ, and stimulate IL-4, IL-10, and IL-13 production [24]. Altogether, this may trigger the development of GD, which is considered to be a Th2-dependent autoimmune disease. Accordingly, the role of psychosocial stress in the etiopathogenesis of GD has been suggested by numerous studies. Stressful life events trigger both the onset and recurrence of hyperthyroidism in GD patients who were followed up for at least 5 years after antithyroid drug withdrawal [25]. Several anecdotal GD cases reported an acute stress event prior to the occurrence of the disease [26]. However, a prospective 5-year follow-up study with 790 participants who had positive family anamnesis for AITD, found no causal association between stress and GD [27]. Thus, the role of stressful events in the development of GD remains controversial.

The relationship between stress and HT has not been thoroughly explored. According to several prospective and cross-sectional studies and observations, stress does not have any triggering role in HT [7,28]. In contrast, a connection between stress and HT was found in other studies, addressing the impact of chronic stress-induced trauma in childhood [29,30,31]. However, it has been hypothesised that in individuals with a susceptibility to thyroid autoimmunity, an enhancement of cell-mediated immune function due to a “return shift” from the Th2 to Th1 immune response could occur following stress [32], so there is a hypothetical mechanism by which stress can contribute to the pathogenesis of HT.

Another point of intersection between psychosocial stress and AITD is the status of selenium, an essential trace element necessary for the synthesis of the amino acid selenocysteine, which is integrated into selenoproteins [33]. The physiological roles of selenoproteins are wide-ranging. The most important examples could be the glutathione peroxidase family and the thioredoxin reductases that protect against oxidative stress damage, endoplasmic reticulum stress, and inflammatory reactions [34]. It is well-established that psychological stress induces neuronal damage and dysfunction via oxidative stress. In addition, during stress, elevated levels of glucocorticoids can downregulate antioxidant enzymes [35], exacerbating the neurobehavioural consequences of stress. On the other hand, the thyroid gland has the highest concentration of selenium in the body. Notably, thyroid hormone synthesis produces an excess of hydrogen peroxide, which can modify antigens and damage thyrocytes [36], thus representing a potential initiating pathway in the pathogenesis of AITD. Therefore, low selenium levels and declining selenoprotein biosynthesis might aggravate the damage to thyrocytes. One of the most reliable parameters of selenium status is selenoprotein P (SeP), which is rich in selenocysteine and comprises approximately 25% of the body selenium, thereby maintaining selenium homeostasis [37,38].

In this study, we aimed to investigate the relationship between the number and impact of stressful life events in AITD patients, the Th1/Th2/Th17 immune response, and the selenium parameters, to better understand the effects of stress-related mechanisms on the immune system.

## 2. Results

The demographic and biochemical characteristics of the participants are presented in Table 1. All three study groups–HT, GD, and controls–were similar in age and gender. Thyroid function (thyroid stimulating hormone (TSH), free thyroxine (FT4),and free triiodothyronine (FT3)) and thyroid autoimmunity (thyroid peroxidase antibody (TPO-Ab), thyroglobulin antibody (Tg-Ab), and TSH-receptor antibody (TR-Ab) level) results showed significant differences between the groups (all *p* < 0.001 and the Kruskal–Wallis test) in accordance with the disease characteristics. Post hoc tests showed significant differences in these parameters between all groups, except FT3 in controls vs. HT (*p* = 0.676), TPO-Ab in GD vs. HT (*p* = 0.964), and Tg-Ab in GD vs. HT (*p* = 0.921). No significant differences in serum selenium (Se), glutathione peroxidase (GPx), or selenoprotein P (SeP) levels were found among the patients with HT, the patients with GD, and the controls (all *p* > 0.05). A low serum selenium, as defined by the selenium level <80 µg/L, was found in 38.8% of the controls, 42.6% of the HT group patients, and 61.5% of the GD group patients. Furthermore, a significant positive correlation between selenium and selenoprotein P was found in the HT patient group (r_s_ = 0.392 and *p* = 0.007). No significant differences in the number of stressful life events were found among the patients with HT, the patients with GD, and the controls; however, negative stress levels tended to be higher in the patients with HT and GD than in the controls (Table 1). Furthermore, a significantly higher negative stress level compared to the positive stress level was found in the HT group (*p* = 0.023), but the difference was not significant in the other study groups (*p* = 0.406 in the control group and *p* = 0.505 in the GD group).

**Figure 1:**
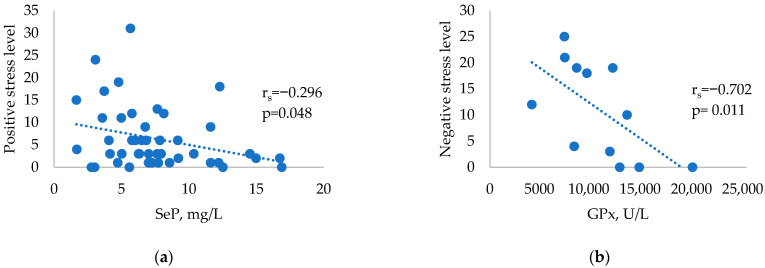
Correlations between selenium parameters and stressful events in HT and GD patients: (**a**) Correlation between SeP and positive stress levels in HT patients. (**b**) Correlation between GPx and negative stress levels in patients with GD.

Further correlations between the number of major life events from the Life Experiences Survey and selenium parameters were analysed within the three study groups. There were no significant correlations in the control group (all *p* > 0.05). The HT group showed significant negative correlations between SeP and positive stress levels (Figure 1a). The GD group showed significant negative correlations between GPx and negative stress levels (Figure 1b).

Plasma levels of Th17-related cytokines, Th17-promoting cytokines, Treg-associated cytokines, Th1-related cytokines, and Th2-related cytokines did not differ significantly between the study groups (Table 2).

**Figure 2:**
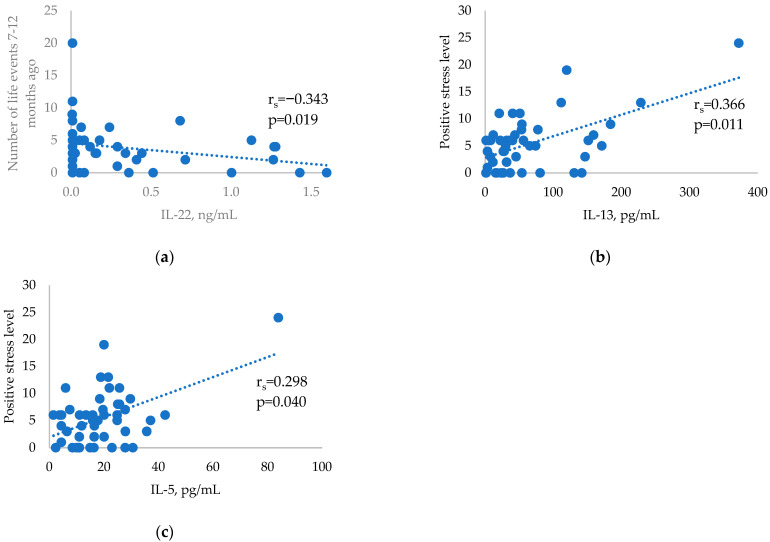
Correlations between selected cytokines and stressful life events in the HT and control groups: (**a**) Correlation between IL-22 and stressful life events occurring 7–12 months before HT diagnosis. (**b**) Correlation between positive stress levels and IL-13 in the control group. (**c**) Correlation between positive stress levels and IL-5 in the control group.

Cytokine levels were analysed in relation to stressful life events in the three study groups and showed no significant correlations in the GD patients (*p* > 0.05). There was a significant negative correlation between the Th17-related cytokine IL-22 and stressful life events 7–12 months prior to the HT diagnosis (Figure 2a). Two significant Th2-related cytokine correlations with positive stress scores were found in the control group (Figure 2b,c).

Thyroid autoantibody level correlations with different parameters of the Life Experiences Survey were also analysed. Significant correlations between various stress parameters and TPO-Ab and Tg-Ab were found (Table 3).

When cytokines IL-13, IL-22, and IL-5 were mutually adjusted in the logistic regression analysis, only IL-22 showed a significant association with GD (*p* = 0.031). The higher level of IL-22 was associated with lower odds of GD (the OR of one unit IL-22 increase was 0.03 (95%CI 0.01–0.73)), which became non-significant after the other factors were added to the model. The total number of major life events was neither a significant factor itself, nor did it affect the strength of the association between cytokines and AITDs. Negative life events were associated with higher odds of HT: the OR associated with one event increase in the number of negative events was 1.10 (95%CI 1.01–1.19). The timing of the stressful life events, in the last six months or earlier, did not have any effect. GPx did not show any association with AITDs, but selenium showed a borderline significant (*p* = 0.05) association with a lower probability of GD: OR 0.98 (95%CI 0.95–1.00). The final model is presented in Table 4.

## 3. Discussion

Chronic stress has been shown to be associated with systemic inflammation, leading to increased proinflammatory cytokine levels, while stress-induced cytokine changes may be implicated in the development of autoimmune diseases, including AITD [39]. Although some authors indicate that only undesirable events lead to psychological impairment [40,41], it can be argued that in the same way, all life changes require adaptation and may be stressful at the individual level. We analysed possible relationships between the number and impact of stressful life events and the plasma levels of several Th1, Th2, and Th17 cytokines in patients with new-onset AITD. No significant differences in the Th1/Th2/Th17/Treg cytokine levels were found among the HT patients, GD patients, and controls. In contrast to AITD, which is an organ-specific autoimmune disorder, the aforementioned cytokines are secreted by a broad spectrum of immune and nonimmune cells throughout the body; therefore, their production is not organ-specific. Recently, after analysing the expression patterns of Th17-associated cytokines within the thyroid tissue, the expression level of IL-17 in thyrocytes was found to be significantly higher in the HT and GD patients than in controls [42]. However, our results provide evidence for a negative correlation between the plasma IL-22 levels and the number of stressful life events that took place 7–12 months prior to a HT diagnosis.

IL-22, a member of the IL-10 family of cytokines, is produced by different types of lymphocytes, including Th17 and Th22 cells, natural killer T cells, and γδ T cells [43]. Although IL-22 has been associated with the development of autoimmune and inflammatory diseases, such as psoriasis and rheumatoid arthritis, it is a cytokine with dual pro- and anti-inflammatory activities. IL-22 maintains the barrier function and hosts the defence against pathogens by inducing the expression of antimicrobial peptides at mucosal surfaces [44]. Recently, IL-22 has been named a potent endogenous suppressor of oxidative and endoplasmic reticulum stress [45]. On the other hand, higher IL-22 serum levels were found in untreated euthyroid HT patients than in those patients with nodular goitre or in healthy subjects [46]. It is well-known that all life event stressors stimulate physiological responses, releasing glucocorticoids and catecholamines, and promoting a shift in the cytokine balance. Glucocorticoids were shown to inhibit IL-22 production in the human and mice group three innate lymphocytes, and this suppression was glucocorticoid-dependent [47]. The correlation between IL-22 levels and stressful life events is complex, and so it requires further investigation.

In addition, positive relationships between IL-13 and IL-5 and life events with positive stress levels were found in the control group, indicating that a positive stress level also affects Th2 proinflammatory cytokine responses. An elevated IL-13 is able to suppress the synthesis of Th1 proinflammatory cytokines, such as TNFα and IFNγ, as well as the production of several macrophage- and monocyte-derived inflammatory cytokines [48]. In addition, IL-13 protects beta-cells against IL-1β-induced apoptosis. For this reason, IL-13 is classified as an anti-inflammatory interleukin [49]. In this study, we also found a negative correlation between GPx and negative stress levels, which was only significant in patients with GD, a predominantly Th2 autoimmune disease. Lower levels of GPx have been described to shift the cytokine production towards the Th2 cytokine response [50], and vice versa, higher levels of GSH induce the differentiation of naïve CD4+ T cells to Th1 cells [51]. Together, these considerations suggest that both positive and negative stress may play a role in the pathogenic mechanism of Th2-mediated diseases.

Although no association between the presence of TPO antibodies and self-reported stress in 759 euthyroid subjects was previously observed in the study by Strieder et al. [28], we found positive correlations between the number of major life events and life events with a negative impact on both TPO and Tg antibody levels, suggesting that stress might be involved in the progression/course of HT. Because the precise onset of the disease is generally unknown, it is difficult to determine the mechanism of stress for triggering HT.

In contrast to GD, the development of HT is mainly associated with the cellular immune responses mediating cytotoxicity and inflammation via increased Th1 and Th17 activity; however, humoral-mediated immune mechanisms are also implicated in the pathogenesis of HT [19]. TPO antibodies have demonstrated several pathogenic effects, such as the complement system activation and the induction of oxidative stress. They are involved in thyrocyte damage by both antibody- and complement-dependent cytotoxicity mechanisms [52]. Moreover, lower antioxidant parameters and higher oxidant parameters were detected in euthyroid untreated HT patients, suggesting that thyroid antibodies are independent risk factors for developing oxidative stress, irrespective of the thyroid function [53]. Selenium, in the form of selenoproteins, has relevant antioxidant properties, and an adequate selenium status not only reduces the level of thyroid antibodies [54,55], but also lowers the risk of AITD [56]. We did not observe any significant differences in plasma selenium, SeP, or GPx levels among the groups, but it is important to highlight that the average selenium status in all groups (87.2 µg/L 71.3 µg/L and 84.6 µg/L) was lower than the recommended optimal level of 125 µg/L [57]. Optimal and effective synthesis of GPx requires a plasma Se level of at least 100 µg/L [58], but concentrations of approximately 120 µg/L are required for the maximal expression of SeP, which reflects the functional selenium pool [59]. Therefore, our data demonstrate selenium concentrations that are considered deficient for optimal antioxidant enzyme expression and function. Various exogenous selenium-containing compounds with immunomodulatory and anti-inflammatory properties are described to contribute to the regulation of diseases of the immune system [60]. Recently, a source of Se that is receiving increased interest is nanoparticles (SeNPs), which have shown potential therapeutic benefits in various diseases mediated by oxidative stress and inflammation systems and have demonstrated the ability to modulate the functional state of neutrophils in mice [61]. Another interesting aspect of our study is the negative correlation between the number of stressful events and the concentration of selenoproteins, which supports the taking of the measurements for selenoprotein levels/activity instead of plasma selenium because the selenium content also includes the functionally inactive selenium incorporated within the protein structure as selenmethionine [62].

Interestingly, the effects of stress management were recently studied in HT patients, and this study demonstrated a significant reduction in the anti-TG antibody titres and stress levels after an 8-week stress management intervention [12]. Although the implementation of stress management in the treatment strategy for HT patients is still questionable, we believe that patients might benefit from such interventions.

## 4. Materials and Methods

This cross-sectional study was implemented at the Riga East University Hospital, from September to December 2020. The study was carried out after receiving approval by the ethics committee of Riga East University Hospital Research Committee and the Central Medical Ethics Committee, Latvia (Decision No. 01-29.1/5033).

This study enrolled 109 adult participants (16 males and 93 females). The inclusion criteria were: (1) newly diagnosed cases of AITD: Hashimoto’s thyroiditis (positive circulating antibodies to thyroid antigens: Thyroid peroxidase antibody (TPO-Ab) and/or Thyroglobulin antibody (Tg-Ab) values greater than 100 IU/mL were considered diagnostic) or Graves’ disease (decreased TSH levels and positive TSH-receptor antibodies (TR-Ab); (2) healthy controls–healthy individuals who do not have the following diseases or special diets: liver or kidney disorders, a vegetarian or vegan diet, any other autoimmune disorder, and a participant positive for antinuclear antibodies (ANA) or tissue transglutaminase IgA (tTg-IgA) antibodies; (3) the patient has not used medications affecting the thyroid function. The exclusion criteria were: (1) pregnancy; (2) if the participant is less than 18 years old; (3) if the patient is taking medications that can affect the thyroid/immune function–Amiodarone, for example, corticosteroids, antidepressants, etc. The study population was divided into 3 groups: Hashimoto’s thyroiditis group (*n* = 47), Graves’ disease group (*n* = 13), and control group (*n* = 49). All participants of the study provided written informed consent before enrolment and had a blood sample collected.

### 4.1. Materials of the Study

Peripheral blood samples were collected from all 109 participants. Thyroid function tests (the levels of serum-free thyroxine (FT4), free triiodothyronine (FT3), and TSH) and the levels of TPO-Ab and Tg-Ab were measured using chemiluminescence immunoassays (Siemens, Malvern, PA, USA). Reference values were as follows: FT4, 0.7–1.48 ng/dL; FT3, 0.2–0.44 ng/dL; TSH, 0.35–4.94 μIU/mL; TPO-Ab, 0–60 IU/mL; and Tg-Ab, 0–40 U/mL. Plasma levels of TR-Ab, ANAs, and tTG-IgA autoantibodies were detected by ELISA (Pharmacia Diagnostics Freiburg, Germany), reference range: TR-Ab, 0–1.58 IU/L; ANA, reference range was “negative”; tTG-IgA, 0–10.0 U/mL. Analyses of clinical variables were performed in an accredited diagnostic hospital laboratory using automated diagnostic equipment.

### 4.2. Plasma Levels of Cytokines

Th17-related cytokines (IL-17a and IL-22), Th17-promoting cytokines (IL-23 and IL-6), Treg-associated cytokines (IL-10), Th1-related cytokines (IFN-γ and IL-2), and Th2-related cytokines (IL-4, IL-5, and IL-13) were analysed in duplicates with the plasma samples in EDTA, and were analysed for immunological markers using xMAP technology (Magpix system, Luminex Corporation, Austin, TX, USA).

### 4.3. The Plasma Selenium

Concentration was determined fluorometrically using a fluorescence spectrophotometer “Cary Eclipse” (Varian, Inc., Houten, The Netherlands). Interlaboratory quality control was conducted by employing two standards—selenium AAS solution (Aldrich, St. Louis, MO, USA, Cat# 24, 792-8) and Seronorm TE Serum Level I (Sero AS, Cat# 201 405, Billingstad, Norway)—for the Seronorm™ Trace Elements-Controls Programme. External Quality Assessment Services were performed by Labquality Oy, Finland.

### 4.4. Determination of Glutathione Peroxidase (GPx) 3 and (EC) 1.11.1.9 Activity

The activity of GPx was evaluated in heparinised whole blood by the conventional method of Paglia and Valentine (50), using commercial tests manufactured by Randox Laboratories (UK, Antrium) in a RXDaytona analyser. GPx from the sample of heparinised whole blood catalysed the oxidation of GSH (4 mmol/L) by cumene hydroperoxide (0.18 mmol/L). Oxidised glutathione (GSSG) was converted to GSH in the reaction catalysed by glutathione reductase (>0.5 U/L) in the presence of NADPH (reduced form of nicotinamide adenine dinucleotide phosphate) (0.34 mmol/L), which led to a decrease in absorbance, which was further measured at 340 nm. One unit corresponds to the amount of enzyme produced by 1.0 μMNADPH oxidation at NADP+1 min at 340 nm at 37 °C, and GPx activity was expressed as U/L of the haemolysate.

### 4.5. Selenoprotein P (SeP)

Concentrations were measured using Spark^®^ (Tecan Group Ltd.) multimode microplate reader by a validated commercial SELENOP-specific ELISA (Cusabio, Wuhan, China) for human cells and a rat selenoprotein P (Selenop) ELISA kit (Cusabio), according to the instructions of supplier.

### 4.6. Stress Parameters

To assess the parameters of stress, participants were asked to fill out the Life Experiences Survey [63] to quantify the emotional impact of important life events. The Life Experiences Survey counts the total number of major life events experienced in the last 12 months (checklist of 47 possible events). The participant separately scored the positive or negative impact of each experienced life event, rated on a scale of zero (meaning no impact at all) to three (significant positive or negative impact). From this scale, the total scores of positive (e.g., outstanding personal achievement) and negative (e.g., death of a close family member) impacts were summarised and thus, the total scores of self-reported psychosocial stresses were calculated. The parameters of self-reported psychosocial stress were both the number of stressful life events (total, 0–6 months ago, and 7–12 months ago) and the score of the self-assessed impact of these life events (positive, negative, and none).

### 4.7. Statistics

For descriptive statistics, the data are presented as the median values and interquartile ranges (IQRs). A nonparametric Kruskal–Wallis test was used to detect the differences between the groups. A post hoc analysis to compare the two independent samples was performed using the Mann–Whitney test. The Wilcoxon signed-rank test was used to compare the sets of scores without a normal distribution that came from the same participants. A nonparametric Spearman’s correlation analysis was performed to determine the relationships of the studied parameters in each subgroup of the study cohort. A multiple logistic regression analysis was used to explore the associations between measures of stressful life events, cytokines, and selenium as the independent factors and the presence of AITDs as the dependent variable. A *p*-value < 0.05 was considered statistically significant for all statistical tests. Statistical analysis of the data was performed in IBM SPSS version 26.0.

## 5. Conclusions

Even though the autoimmune thyroid disease patients did not differ in the number of stressful life events compared to the control group, a significantly higher negative stress level was found in the Hashimoto’s thyroiditis group. In addition, the thyroid peroxidase antibody level correlated with the number of stressful life events, providing evidence for stress-induced changes in immune pathways. We suggest measurements of Th2-related cytokines and selenoproteins as biomarkers for the development of autoimmune thyroid disease in cases where stress is considered a component cause of the pathogenic mechanism of the disease.

## Figures and Tables

**Table 1:** Demographic and biochemical characteristics of the study groups. Numerical data presented as median (interquartile range).

	HT Patients (*n* = 47)	GD Patients (*n* = 13)	Controls (*n* = 49)	*p* Value
Sex (female/male)	44/3	11/2	38/11	0.072
Age (years)	41 (27–50)	41 (29–57)	30 (26.5–46.5)	0.247
TSH (μIU/mL)	2.19 (1.47–3.89)	0.0001 (0.0000–0.0004)	1.12 (0.88–1.75)	<0.001
FT4 (ng/dL)	0.91 (0.86–0.99)	1.97 (1.75–2.55)	0.98 (0.92–1.07)	<0.001
FT3 (ng/dL)	0.32 (0.29–0.34)	1.21 (0.98–2.20)	0.32 (0.30–0.34)	<0.001
TPOAb (IU/mL)	293.04 (125.77–530.59)	215.12 (22.63–1093.52)	0.46 (0.21–0.80)	<0.001
TgAb (U/mL)	<20 ^#^ (<20 ^#^–45.28)	<20 ^#^ (<20 ^#^–146.80)	<20 ^#^	-
TR-Ab (IU/L)	<1.58	12.13 (4.30–24.54)	<1.58	-
Se (µg/L)	93.19 (71.64–118.65)	71.33 (58.92–104.38)	90.93 (71.64–118.65	0.287
GPx (U/L)	12827.5 (10191.5–15006)	10571.5 (7526.75–13194.5)	12962 (9350–14792)	0.246
SeP (mg/L)	6.92 (4.94–9.50)	5.79 (4.52–7.71)	6.41 (4.37–7.85)	0.315
Total number of major life events	7.5 (5.75–11)	7 (4.5–16)	7 (3–9)	0.355
Number of major life events in the last 0–6 months	4 (2–7)	4 (1.4–6)	3 (1–6)	0.477
Number of major life events in the last 7–12 months	3 (1–5)	3 (0–13)	2 (0.25–4.75)	0.348
Number of major life events with no impact	0 (0–1.25)	0 (0–2)	0 (0–1)	0.209
Negative stress level	7.5 (4–12.25)	10 (1–19)	6 (3–8)	0.103
Positive stress level	3 (1–9.5)	5 (2–12.5)	5 (1.25–7)	0.664

^#^ Below limit of detection.

**Table 2:** Plasma levels of cytokines.

	HT Patients (*n* = 47)	GD Patients (*n* = 13)	Controls (*n* = 49)	*p*
IFN-γ (pg/mL)	24.47 (18.74–37.49)	22.75 (14.99–32.55)	20.55 (9.71–37.63)	0.343
IL-10 (pg/mL)	9.25 (5.94–15.21)	8.70 (5.84–10.51)	8.80 (5.37–15.39)	0.761
IL-13 (pg/mL)	33.26 (19.42–60.92)	23.49 (16.97–59.69)	36.90 (16.58–79.21)	0.577
IL-17a (pg/mL)	12.95 (9.16–19.61)	12.37 (9.82–16.43)	11.79 (5.96–18.55)	0.530
IL-22 (ng/mL)	0.12 (0.00–0.51)	0.14 (0.00–0.32)	0.37 (0.15–0.57)	0.063
IL-2 (pg/mL)	0.22 (0.09–0.38)	0.11 (0.07–0.27)	0.26 (0.09–0.42)	0.164
IL-4 (ng/mL)	0.31 (0.17–0.64)	0.27 (0.18–0.63)	0.37 (0.10–0.78)	0.821
IL-23 (ng/mL)	2.83 (1.43–4.94)	2.31 (1.81–3.27)	3.42 (2.08–4.83)	0.357
IL-5 (pg/mL)	15.89 (11.00–25.98)	14.53 (11.28–22.84)	16.51 (9.50–24.99)	0.783
IL-6 (pg/mL)	6.58 (0.00–31.50)	3.66 (0.00–20.36)	11.28 (1.29–49.09)	0.345

**Table 3:** Correlations between various stress parameters and TPO-Ab and Tg-Ab.

	TPO-Ab Level		Tg-Ab Level	
	Spearman’s Correlation Coefficient	*p* Value	Spearman’s Correlation Coefficient	*p* Value
Total number of major life events	0.220	0.023	0.216	0.039
Number of major life events in the last 0–6 months	0.117	0.231	0.134	0.204
Number of major life events in the last 7–12 months	0.211	0.029	0.124	0.241
Number of major life events with no impact	0.228	0.018	0.187	0.074
Negative stress levels	0.218	0.024	0.215	0.039
Positive stress levels	0.100	0.304	0.050	0.636

**Table 4:** Odds ratio of AITDs in association with selected cytokines, the number of negative major life events, and serum selenium: multiple regression model.

	HT vs. Controls		GD vs. Controls	
	OR (95%CI)	*p*	OR (95%CI)	*p*
IL-13 (pg/mL)	0.99 (0.98–1.00)	0.103	0.99 (0.96–1.01)	0.288
IL-22 (ng/mL)	1.66 (0.93–2.99)	0.088	0.05 (0.01–1.12)	0.059
IL-5 (pg/mL)	1.05 (0.99–1.11)	0.115	1.11 (0.97–1.27)	0.130
No. of major life events with negative impact	1.10 (1.01–1.19)	0.028	1.10 (0.99–1.23)	0.088
Se (µg/L)	1.00 (0.98–1.01)	0.551	0.98 (0.95–1.00)	0.050

## Data Availability

The datasets used and/or analysed during the current study are available from the corresponding author on reasonable request.
